# Sustaining the implementation of alcohol management practices by community sports clubs: a randomised control trial

**DOI:** 10.1186/s12889-019-7974-8

**Published:** 2019-12-10

**Authors:** Tameka McFadyen, Luke Wolfenden, Melanie Kingsland, Jennifer Tindall, Shauna Sherker, Rachael Heaton, Karen Gillham, Tara Clinton-McHarg, Christophe Lecathelinais, Bosco Rowland, John Wiggers

**Affiliations:** 1 School of Medicine and Public Health, The University of Newcastle, University Dr, Callaghan, NSW 2308 Australia; 2 Hunter New England Population Health, Booth Building, Longworth Ave, Wallsend, NSW 2287 Australia; 3 Alcohol and Drug Foundation, 607 Bourke St, Melbourne, VIC 3051 Australia; 4 School of Psychology, Deakin University, Burwood Hwy, Burwood, VIC 3125 Australia

**Keywords:** Alcohol, Risky drinking, Football, RCT, Web-based support, Sustainability

## Abstract

**Background:**

Risky alcohol consumption is responsible for a variety of chronic and acute harms. Individuals involved in organised sport have been identified as one population group who consume risky amounts of alcohol both at the elite and the non-elite level. ‘Good Sports’, an alcohol management intervention focused on the community sports setting has been successful in addressing risky alcohol use and alcohol-related harm amongst players and sports fans. Sustaining such implementation effects is a common challenge across a variety of community settings. The primary aim of this trial was to assess the effectiveness of a web-based program in sustaining the implementation of best-practice alcohol management practices by community football clubs, relative to usual program care (i.e. control clubs).

**Methods:**

Non-elite, community football clubs in the Australian states of New South Wales and Victoria, that were participating in an alcohol management program (Good Sports) were recruited for the study. Consenting clubs were randomised into intervention (*N* = 92) or control (*N* = 96) groups. A web-based sustainability intervention was delivered to intervention clubs over three consecutive Australian winter sports seasons (April–September 2015–2017). The intervention was designed to support continued (sustained) implementation of alcohol management practices at clubs consistent with the program. Control group clubs received usual support from the national Good Sports Program. Primary outcome data was collected through observational audits of club venues and grounds.

**Results:**

A total of 92 intervention clubs (574 members) and 96 control clubs (612 members) were included in the final analysis. At follow-up, sustained implementation of alcohol management practices was high in both groups and there was no significant difference between intervention or control clubs at follow-up for both the proportion of clubs implementing 10 or more practices (OR 0.53, 95%CI 0.04–7.2; *p* = 0.63) or for the mean number of practices being implemented (mean difference 0.10, 95%CI -0.23-0.42; *p* = 0.55). There were also no significant differences between groups on measures of alcohol consumption by club members.

**Conclusions:**

The findings suggest that sustained implementation of alcohol management practices was high, and similar, between clubs receiving web-based implementation support or usual program support.

**Trial registration:**

Australian New Zealand Clinical Trials Registry ACTRN12614000746639. Prospectively registered 14/7/2014.

## Introduction

Each year there are more than three million deaths and over 200 varieties of disease and injuries worldwide attributable to harmful alcohol use [1]. Individuals involved in organised sport have been identified as one population group who consume risky amounts of alcohol both at the elite and the non-elite level [2–7]. It is well reported that young males and those involved in contact team sports have high levels of risky alcohol consumption [3, 5, 8]. Additionally, higher levels of alcohol-related harm have been reported among players and spectators of male-dominated, team and contact sports, compared to those not associated with sport [7, 9–11].

The community sports setting has been well cited as an opportune setting to effectively implement alcohol management programs which address risky alcohol use and alcohol-related harm amongst players and sports fans [12]. For example, a randomised controlled trial conducted in community sports clubs in Australia found a significant increase in the implementation of alcohol management practices (e.g. the provision of low-alcohol drink options, sale of substantial food when alcohol is sold and safe transport policies) as part of an alcohol harm reduction program (‘Good Sports’) in intervention clubs (38%) compared to control clubs (25%) [13]. The trial also resulted in a significant decrease in the proportion of intervention club members who engaged in risky alcohol consumption at the club (19%) and were at risk of alcohol-related harm (38%) compared to control club members (risky drinking: 24%; risk of alcohol-related harm: 45%) post-intervention [14]. In Australia, sports clubs are not permitted to sell alcohol without a state or territory specific liquor licence or permit. Liquor licensing requirements differ between each state and territory within Australia, however there are some similarities, including legal drinking age, sale of alcohol to under 18 s and alcohol labelling [15]. To ensure an ongoing contribution to the health of the community, it is important that the implementation of effective interventions are sustained [16, 17]. Achieving sustained implementation effects of health promotion programs has been found to be a challenge across a variety of community settings [18, 19]. For example, a review of community-based health-related programs in the United States and Canada (*N* = 17) found that only 29% (*n* = 5) achieved sustainability of implementation of at least one program component post-intervention for more than 80% of their sites [19]. To date, no studies conducted within the sports club setting have reported data outlining the sustainability of improvements in alcohol management practices. Effective and efficient mechanisms to support sustained program implementation need to be identified, with particular consideration given to the challenges of implementation at scale, across large populations and geographic areas [20]. In the absence of clear guidance from empirical studies on sustainability interventions in this setting [21], the use of theoretical frameworks can provide a useful guide for development of such interventions. For example, The Sustainability Framework [22, 23], suggests that intervention sustainability can be facilitated through the use of strategies to improve strategic planning, environmental support, organisational capacity, communication, partnership and program adaptation.

The use of web-based programs to support ongoing implementation of program elements is one possible solution to the logistical challenges of program sustainability in sports and other settings. The potential exists for web-based programs to be delivered to large numbers of sporting clubs across large geographic areas at relatively low cost. While the use of web-based programs to support implementation or quality improvement initiatives have been well cited in other settings such as hospitals, general health care, [24, 25] and schools, [26–28] there is limited evidence assessing the effectiveness of these programs in supporting sports clubs to maintain adherence to alcohol management practices. The primary aim of this trial was therefore to assess the effectiveness of a web-based program in sustaining the implementation of best-practice alcohol management practices by community football clubs (i.e. intervention clubs), relative to their usual care (i.e. control clubs). A secondary aim of the trial was to assess the impact of the program on alcohol consumption among members of community football clubs.

## Methods

### Design and setting

A randomised controlled trial was conducted with community football clubs within regional areas of New South Wales (NSW), Australia and throughout metropolitan and regional areas of the state of Victoria, Australia. Clubs were either randomised into a ‘web-based sustainability program’ (i.e. intervention) or a ‘minimal contact control’ (i.e. control) group.

### Participant eligibility

#### Football clubs

Non-elite, community-level football clubs that were participating in an alcohol harm-reduction program (Good Sports) were recruited to participate in the trial. Good Sports is a preventative health program in which clubs progress through three levels of alcohol management accreditation [29].

Football clubs were eligible to participate in the study if they; 1) had held the highest level of accreditation (Level 3) within the Good Sports program for a minimum of 12 months; 2) self-reported the implementing of at least 80% of the alcohol management practices required of Level 3 Good Sports clubs; 3) were a non-elite, community-level football club; 4) were an Australian Football League, Rugby League, Rugby Union or Soccer club; 5) had a current valid liquor licence; 6) sold alcohol; 7) had at least one senior (over 18’s) team; and 8) had access to the internet.

#### Football club members

Current club members or affiliates (including players, committee members, spectators and coaches) of participating sporting clubs, who spoke English and were at least 18 years old, were eligible to participate in data collection for the secondary outcome.

### Recruitment procedures

#### Football clubs

A list of Level 3 football clubs and executive committee members for each club was generated using Good Sports program records. Study information and participation sheets were sent via postal mail to a club executive member, (e.g. club president, vice president or secretary) of each club. Club representatives were contacted via telephone 2 weeks after the information was sent, to screen the club for eligibility and assess interest in study participation. Contact details were confirmed and new information sheets were sent via email or postal mail where necessary. Follow-up phone calls continued until club representatives were able to make a decision about their club’s participation in the study, which would often involve a discussion with the club management committee. If club representatives were unable to be contacted, alternative contacts were sought using program databases, relevant football code association websites and other publicly available forums. Consenting clubs were asked to complete and sign a consent form and return it to the research team. A dedicated member of the research team managed all recruitment procedures.

#### Football club members

For the purpose of participating in data collection for the secondary outcome, club members were recruited at club grounds during a senior home game using a pilot-tested, standardised recruitment protocol that detailed specified locations (e.g. club bar or main alcohol service area) for recruitment. A different cohort of club members were recruited at baseline and follow-up. A computer program generated a random number sequence and integrated it into the data collection tool. The random number sequence identified the order in which members who walked past recruiters at the grounds were to be approached by the research staff. Research staff would assess eligibility, provide the study information sheet and invite members to participate. Research staff collected contact details for eligible and consenting club members and continued until up to 20 club members were collected. Consenting club members were contacted in a random order via a phone call from the research team, formally inviting them to participate in the study. Phone calls continued until at least five members from each participating club completed the study survey.

#### Random allocation and blinding

After baseline data collection, clubs were randomly allocated to either the intervention or control group by an independent statistician using a computerised random number function in a 1:1 ratio. Stratification by sports code was undertaken as part of the randomisation procedure, as was previously done by the research team [14] and others, [30], to account for the demonstrated association between sports code and outcomes pertaining to risky alcohol consumption and alcohol-related harm. Stratification by state (NSW or Victoria) was also undertaken due to operational differences between football clubs in these jurisdictions. Research assistants who collected trial outcome data via field observations were blinded to the allocation of clubs to intervention or control groups. The effectiveness of this blinding was tested by asking research assistants to guess the group allocation of clubs following post intervention data collection. Research assistants correctly guessed group allocation for 47% of intervention clubs and 52% of comparison clubs. This was an open trial with club representatives informed of the treatment status of their club following pre-test data collection due to the difficulty in blinding clubs to their allocated group.

#### Alcohol management practices

Intervention clubs were supported to maintain the implementation of the alcohol management practices previously targeted by the club’s involvement in the Good Sports Program (see Table 1). These alcohol management practices are consistent with legislation and guidelines regarding the sale and supply of alcohol in licensed premises [31–34] and have been found to be associated with lower levels of alcohol consumption in licensed premises, broadly, [35, 36], and community sports clubs, specifically [37, 38].

**Table 1:** Alcohol management practices

Practice	
Substantial food provided when alcohol sold	
Non-alcoholic drinks 10% cheaper than full strength alcoholic drinks	
Drunk/intoxicated people not allowed to enter club	
Low-alcoholic drinks 10% cheaper than full strength alcoholic drinks	
Four non-alcoholic options available for purchase	
People under 18 years of age do not serve alcohol	
Drunk/intoxicated people not served alcohol	
Drunk/intoxicated people not permitted to remain on club premises	
Free water provided when alcohol sold	
Staff do not consume alcohol whilst on duty	
One low-alcoholic drink option available	
Licensing signs visible at all bars	
No drink promotions undertaken at the club (happy hour, all you can drink functions, alcohol-only awards and prizes, cheap drinks, drinking games, drinking vouchers/cards)	

#### Web-based sustainability intervention

A web-based sustainability intervention was delivered to intervention clubs over three consecutive Australian winter sports seasons (April–September 2015–2017). An expert advisory group consisting of community sports club representatives, health promotion practitioners, and experts in community organisational change and reducing alcohol-related harm associated with licensed premises, developed the program based on implementation and behaviour change theory [23, 39] and evidence [13]. A web-based intervention was selected due to the efficiency it presented in: delivering the program to the large number of sports clubs located across the geographic spread of the two states; the low-cost of maintaining and updating the program centrally; and the flexibility in tailoring the intervention to clubs individual needs. Such computer-based interventions have been used to improve and sustain the health promoting practices of organisations [40]. The intervention was designed based on theoretical frameworks for sustainability (The sustainability framework [22, 23]) and behaviour change (The Persuasive Design framework [39]). The intervention strategies are outlined in Table 2 and are mapped against the key domains from these conceptual frameworks. A full description of the intervention can be found in the published protocol paper [41].

**Table 2:** Intervention strategies and conceptual frameworks

Intervention strategy	Description	The Sustainability Framework construct [35]	Persuasive Systems Design Framework construct [36]
Club champion	• Club champions were members of the club executive (president, vice president, treasure, secretary) • Club champions primarily engaged with the web-based program on behalf of the club to complete the annual online assessment and action plan.	Environmental support Organisational capacity	
Executive support	• Each year, clubs were encouraged to have the alcohol management policy reviewed at the club’s committee meetings by all executive members • Results from the club’s annual online assessment and action plan were automatically emailed to club executive members.	Environmental support Organisational capacity Strategic planning	
Targeting interactive intervention	• Clubs had access to their own club portal where they could update their club contact details and select current annual online assessment or action plan • A tailored action plan was automatically developed based on responses from the annual online assessment • Links to appropriate options/strategies and resources were available for all required actions • Clubs were able to adjust the suggested completion dates for generated action items (within reasonable limits) • Clubs were able to track their own progress by updating the status of action items (not started/in progress/complete).	Program adaptation	Tunnelling Tailoring/ personalisation Self-monitoring
Tailored feedback	• Clubs received tailored feedback both within the web-based program and via email, based on their completion of the annual online assessment and completion of agreed actions.	Program adaptation	Tunnelling Tailoring/personalisation
Training and support	• For any user problems help options were available via email or phone.	Organisational capacity	Ease of use/ accessibility
Tools and resources	• Printable instructional materials, sample policies and planning templates were available via the web-based program	Organisational capacity	Tailoring/ personalisation
Systems and prompts	• Email reminders were sent to: prompt annual online assessment completion and when action plan items due dates are approaching or are overdue.	Environmental support	Reminders and prompts Tailoring/ personalisation
Communication and marketing	• An independent nominee (a community stakeholder) was be sent an email from the program each time their relevant club completed the annual assessment informing them of the club’s progress.	Communication Partnership	Praise Rewards Recognition
Recognition and reward	• Automatic notification and praise via emails were sent to club champions and club executives when the annual online assessment and action plan was complete. • E-newsletter acknowledged club progress in comparison to peers.		Praise Rewards Recognition

Each year intervention clubs were supported to undertake an online assessment of club alcohol management practices and online action planning based on identified practice needs. Sports clubs would first engage with the web-based program via an email invitation which was sent to the club champion at the start of each sporting season within the intervention period. The email included a link to log onto the program directly and the club champion could update their details, and those of the club executive. They would then be prompted to complete the club’s online assessment. The online assessment generated a club specific action plan. Clubs were required to complete the items on the action plan each sporting season. The club champion had the ability to save and exit the assessment and action plan as needed. These could then be updated and completed at a time which suited the club champion. Each year, the results from the club online assessment and action plan would be sent to all executive club members. The club champion also had access to a tools and resources tab and frequently asked questions tab to support their progression and completion of the program steps. Intervention clubs were required to complete a new online assessment and action plan each year, regardless of whether they had completed them the previous year or not, and had a maximum of 3 years of web-based intervention access.

A member from the program team contacted intervention clubs up to four times via telephone to prompt and support annual assessment and action plan completion. Clubs also received email prompts for task completion, confirmation emails when they completed the annual assessment and action plans and up to five e-newsletters.

#### Control group clubs

Control group clubs did not have access to the web-based program or any of the web-based resources. Control clubs received usual support provided to clubs accredited as Level 3 clubs with the Australian Drug Foundation’s Good Sports program [29] which consisted of one phone contact initiated during the 2016 sporting season of the intervention period. Reactive support was provided on an as-needs basis in instances where a club made contact for further support about an alcohol-related incident or concern.

### Data collection procedures

#### Club and club member characteristic data

The operational characteristics of clubs and demographics of sports club members were collected during a telephone survey using items used in previous trials in this setting conducted by the research team.

#### Implementation of alcohol management practices

Club implementation of alcohol management practices was collected via gold standard field observation [42] at baseline (April – August 2015) and follow-up (May – July 2017) by trained research assistants. Research assistants undertook 1 day training sessions facilitated by the research team and were required to successfully complete simulated scenarios of alcohol observation practice prior to undertaking the field observations. The scenarios were a combination of images of potential sports club settings and interactions with members of the research team playing the role of club staff. At the completion of the scenarios, the research team conducted a group consensus process where all research assistants went through their responses, and any discrepancies were discussed, and correct responses were identified.

The field observations occurred at a participating clubs home ground during a senior game. Clubs were not informed of the exact date of the observation visit. Two research assistants undertook the observation at each club, conducting their observations of alcohol management practices independently and comparing complete observation reports at the end of the visit. Upon completion of the observation visit any discrepancies were discussed. If consensus was needed to be reached, research assistants were instructed to check objective observation evidence for alcohol management practices, such as, visible licensing signs at the bar or identifying drunk/intoxicated person who was allowed to remain on club premises. If consensus could not be reached due to the objective evidence being no longer available to observe (ie dunk/intoxicated person had left the premises after the game), research assistants were instructed to record the practice outcome in accordance with the item being observed. Protocols and methods for such observations have been successfully implemented by the research team during more than 200 observations of sports club, hotels and night-clubs as part of previous trials [43, 44]. Pilot testing was conducted with four clubs prior to commencement of baseline data collection to refine the tool.

#### Risky consumption and alcohol related harm

Alcohol consumption at the club was collected via a Computer Assisted Telephone Interview with a repeat cross-sectional sample of club members recruited from the field observations at baseline (2015) and follow-up (2017).

#### Intervention use

Intervention club use and engagement with the intervention website was collected via reports generated through a web-based software system.

### Measures

#### Club and club member characteristics

Club representatives provided data on; number of senior (18 years of age and over) and junior (under 18 years of age) teams and members registered with the club, football code and postcode of the club. Club members provided information on their role/association with the club, age, gender, education and income.

#### Club implementation of alcohol management practice

The primary outcomes of the trial were

i) The proportion of clubs maintaining the implementation of ≥10 of the 13 required alcohol management practices (Table 1). The target of more than 10 practices was selected as it represents approximately 80% or more of intervention practices, a benchmark suggested as more appropriate for assessing implementation fidelity, and which has been applied in other implementation studies [45, 46]. Perfect implementation is considered unrealistic in real world implementation contexts [47].
ii) The mean number of alcohol management practices implemented at follow up, assessed through direct observation at sporting clubs during game days.

The proportion of clubs implementing each of the 13 individual alcohol management practices were included as secondary implementation outcomes. This outcome was not prospectively registered but was included in the published protocol submitted prior to follow up data collection.

#### Risky consumption and alcohol related harm

Secondary outcomes also included:

1. The proportion of club members who reported drinking alcohol at risky levels at sporting clubs as measured by the graduated frequency index [48]. Risky drinking was defined as five or more standard drinks of alcohol on one drinking occasion at least once a month at the club. Five or more drinks were chosen as the cut off as National Health and Medical Research Council Australian Drinking guidelines for the reduction of harm from alcohol consumed on a single occasion recommend drinking no more than four standard drinks on a single drinking occasion [49].
2. The proportion of club members who reported being at-risk of alcohol-related harm, as measured by a total Alcohol Use Disorder Identification Test (AUDIT) score of ≥8 [50]; and,
3. Mean AUDIT score of club members. This outcome was not prospectively registered but was included in the published protocol submitted prior to follow up data collection.

#### Intervention use

Measures used to describe club intervention engagement include the proportion of intervention group clubs that; logged into the intervention site per season; completed the annual assessment per season, and completed or partially completed the club specific action plan per season.

#### Sample size calculations

Assuming at follow-up that 70% of clubs in the control group maintained 80% of the required alcohol management practices (Table 1); that is, the prevalence of adequate practice implementation in the control group would fall from 100% at baseline to 70% at follow-up, a sample size of 60 clubs per group was calculated to be sufficient to detect an absolute difference of 20% between groups at follow-up, with 80% power and alpha of 0.05, as well as detecting a difference of 51.6% of a standard deviation in the mean number of practices required.

### Statistical analysis

Analyses were performed under an intention to treat approach using the statistical software SAS v9.3. The primary trial outcomes were assessed by examining between group differences at follow-up in the: 1) prevalence of clubs maintaining ≥10 of the 13 required alcohol management practices and 2) the mean number of practices being implemented by clubs. Individual practice implementation outcomes were also assessed for between group differences in prevalence at follow-up. For dichotomous outcomes, the difference between the groups was assessed using multiple logistic regression models. For continuous outcomes, differences between groups were assessed using multiple linear regression models. The analyses of each primary and secondary trial outcome controlled for baseline outcome values. Outcome analysis was first performed using complete case analysis, using all available data without imputation. Analyses were also performed using multiple imputation methods, imputing data for any individual practice that was missing for clubs at baseline or follow-up (e.g. practices that assessors did not record) or, imputing all practice data for clubs missing or ineligible at follow-up. Additionally, and for exploratory reasons we completed a per-protocol analyses on the primary outcomes where the effects of the intervention were compared among intervention clubs that received a full intervention dose (i.e. generated and completed an action plan for all three intervention years) compared with those that did not. The per-protocol analyses was not pre-registered. Statistical tests were two tailed and with an alpha of 0.05. Mixed effect logistic regression models were used on dichotomous member level outcomes to measure the difference between group risky alcohol consumption and related harm. A linear mixed effect regression model was be used to assess difference between the continuous member level outcome. Multiple imputation was also performed on member level outcomes of alcohol consumption. If a club had no follow-up data or insufficient (under 5) member data, then artificial members were generated, and multiple imputation was then performed on the member level outcomes through the multiple imputation procedure (MI) in SAS. Exploratory per-protocol analyses was also undertaken on member level outcomes to describe differences in measures among members of the intervention group of clubs that had received a full dose of the intervention with those that had not. This analysis was not pre-specified.

## Results

### Club recruitment and characteristics

**Fig. 1:**
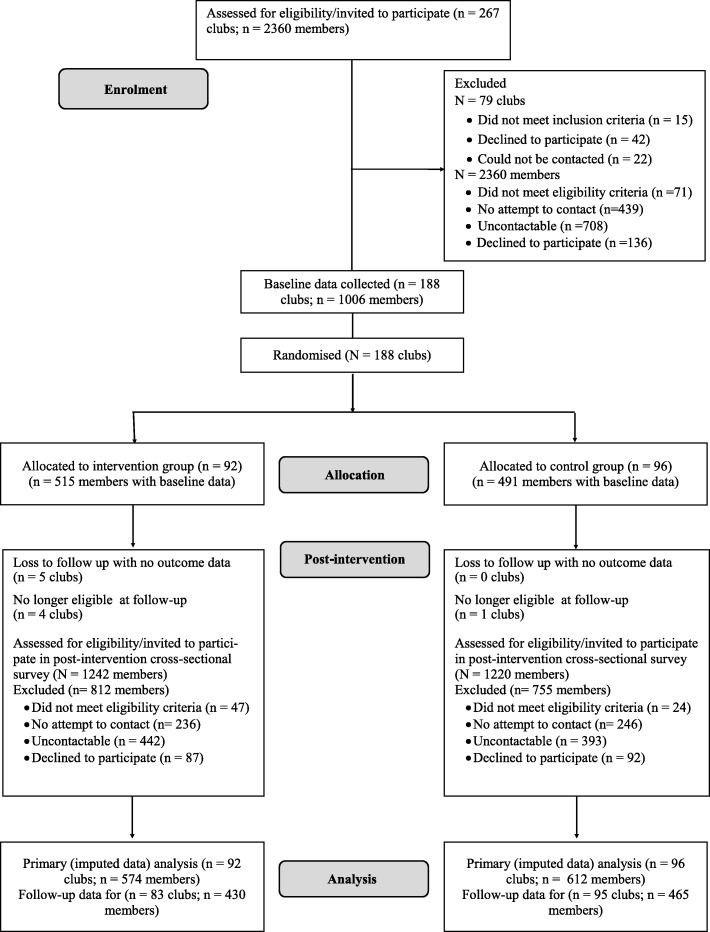
Participant flow according to CONSORT reporting requirements for randomised trials

Two hundred and sixty-seven clubs were identified and contacted within the study area. Of those, 230 were eligible and 188 consented and provided baseline data (Fig. 1). The primary reason for ineligibility was that clubs were no longer selling alcohol. Consenting clubs did not differ from non-consenting clubs in terms of football code [χ^2^ = 6.71; (df) = 4; *p* = 0.15] and geographical location [χ^2^ = 1.10; df = 1; *p* = 0.29]. Consenting clubs were randomised into intervention (*N* = 92) or control (*N* = 96) groups. There was minimal variance between groups in baseline characteristics of clubs and club members as outlined in Table 3. The majority of clubs in both groups were from the Australian Rules football code, located in major cities and classified as small clubs. Follow-up data collection was completed on 178 clubs (83 intervention; 95 control). Five clubs were found to be no longer eligible to participate in the trial as they were not selling alcohol and five withdrew from the study. Of the 10 clubs that did not complete follow-up data collection, 4 were soccer clubs (3 intervention, 1 control), 3 were rugby league clubs (all intervention) and 3 were AFL clubs (all intervention), and, 5 of the 10 were from a major city (4 intervention, 1 control), and 5 of the 10 were classified as small clubs (all intervention). Where data was imputed, there were 92 intervention clubs and 96 control clubs.

**Table 3:** Baseline characteristics

Characteristics	Intervention	Control
*Clubs*	*N* = 92	*N* = 96
*Football code*		
Australian Rules	70%	68%
Rugby League	10%	6%
Rugby Union	4%	6%
Soccer/association football	16%	20%
*Geographical region*		
Major city	64%	51%
Inner/outer regional	36%	49%
*Club size*		
Small (≤ 160 players)	56%	55%
Large (> 160 players)	44%	45%
*Club members who participated in baseline survey*	*N* = 515	*N* = 491
*Club role/association*		
Player	17%	17%
Non playing member (supporter)	57%	61%
Club committee member	23%	21%
Coach/Umpire/referee	10%	9%
Other	22%	20%
*Age of members*		
Mean (SD)	47 (14.01)	47 (14.33)
*Gender*		
Male	71%	70%
Education		
University level	27%	29%
Income		
More than AU$52000	59%	60%

### Club member recruitment and characteristics

During the baseline observation period, 4613 people were randomly selected and approached at the sports club ground, of which 2360 provided their contact details. Of those members that provided contact details: 71 were further assessed during the survey to be ineligible (i.e. they were not a current member or supporter of the club); 136 declined to participate in the survey; 708 were uncontactable and 439 were not contacted as the number of completions per club had already been reached. The remaining 1006 members completed the baseline survey (Fig. 1). At baseline club members from both intervention and control clubs were a mean age of 47 years and the majority identified as non-playing member or supporters and were male as outlined in Table 3. Where data was imputed, there were 574 members from intervention clubs and 612 members from control clubs.

### Club implementation of alcohol management practices

The primary outcomes for the trial are outlined in Table 4. For multiple imputation analysis there was no significant difference between intervention or control clubs at follow-up for both the proportion of clubs implementing 10 or more practices (OR 0.53, 95%CI 0.04–7.2; *p* = 0.63) or for the mean number of practices being implemented (mean difference 0.10, 95%CI -0.23-0.42; *p* = 0.55). There was also no difference found for complete case analysis at follow-up (*p* = 0.39 and p = 0.63 respectively). Among intervention clubs there were no significant differences identified between those clubs which received a full intervention dose (*N*=6) and those which did not, in the proportion of clubs implementing 10 or more practices (OR 0.47, 95%CI 0.07-∞; *p* = 1) or for the mean number of practices being implemented (mean difference 0.73, 95%CI-0.36-1.83; *p* = 0.19) as part of the per-protocol analyses.

**Table 4:** Club implementation of alcohol management practice

Measures	Intervention		Control		Complete cases *N* = 178		Multiple imputation *N* = 188	
	*Baseline* *N = 92*	*Follow-up* *N = 83*	*Baseline* *N = 96*	*Follow-up* *N = 95*	*OR [95%CI]*	*p-value*	*OR [95%CI]*	*p-value*
10 or more practices *% (n)*	89.1% (82)	95.2% (79)	86.5% (83)	91.6% (87)	1.73 [0.50,6.03]	0.39	0.53 (0.04,7.20)	0.63
					*Mean difference [95%CI]*	*p-value*	*Mean difference [95%CI]*	*p-value*
Mean number of practices *Mean (SD)*	11.2 (1.54)	11.6 (1.37)	11.2 (1.59)	11.4 (1.30)	0.09 [−0.29,0.47]	0.63	0.10 (− 0.23,0.42)	0.55

### Implementation of individual alcohol management practices

Table 5 reports the individual practices implemented at baseline and follow-up by trial groups. There were no significant differences between groups in the implementation of any of the targeted individual practices at follow-up. However, two practices (low-alcoholic drinks 10% cheaper than full strength alcoholic drinks and free water provided when alcohol sold) were approaching significance (*p* = 0.06).

**Table 5:** Individual practice implementation

Practice	Intervention		Control			
	Baseline *N* = 92%(n)	Follow-up *N* = 83%(n)	Baseline *N* = 96%(n)	Follow-up *N* = 95%(n)	OR [95%CI]	*p*-value
One low-alcohol drink option available	84 (78)	81 (77)	77 (74)	77 (64)	0.54 [0.24,1.24]	0.15
Four non-alcoholic options available	93 (85)	95 (90)	95% (91)	92 (77)	0.75 [0.22,2.60]	0.65
Low-alcoholic drinks 10% cheaper than full strength alcoholic drinks	89 (70)*	91 (59)*	92 (71)*	83 (65)*	8.02 [0.92,69.55]	0.06
Non-alcoholic drinks 10% cheaper than full strength alcoholic drinks	98 (83)*	100 (81)*	100 (92)	100 (92)*	–	–
Substantial food is provided when alcohol sold	100 (92)	100 (80)*	99 (95)	99 (87)*	0.92 [0.05,∞]	1.00
Drunk/intoxicated people not permitted to remain on club premises	87 (80)	94 (78)	88 (84)	98 (93)	0.33 [0.06,1.73]	0.19
Drunk/intoxicated people not allowed to enter club	98 (90)	98 (81)	96 (92)	100 (95)	0.36 [0–3,08]	0.44
Drunk/intoxicated people not served alcohol	90 (83)	95 (79)	92 (88)	100 (95)	0.17 [0.00,1.00]	0.10
Bar servers do not consume alcohol while on duty	75 (69)	95 (78)	86 (83)	93 (88)	1.78 [0.49,6.48]	0.38
Free water provided when alcohol sold	84 (77)	84 (70)	83 (80)	72 (68)	2.07 [0.98,4.37]	0.06
Licensing signs visible at all bars	73 (67)	67 (56)	70 (67)	61 (58)	1.30 [0.70,2.43]	0.40
People under 18 years of age do not serve alcohol	96 (88)	99 (82)	94 (90)	98 (93)	1.79 [0.16,20.16]	0.64
Club does not permit/conduct the following promotions:						
Happy hour (Cheap or discounted alcoholic drinks)	95 (87)	98 (81)	95 (91)	100 (95)	0.38 [0,3.23]	0.46
Drinking games	100 (92)	100 (83)	99 (95)	100 (95)	–	–
Alcohol-only player awards	100 (92)	100 (83)	100 (96)	100 (95)	–	–
Alcohol-only prizes	99 (91)	96 (80)	96 (92)	98 (93)	0.44 [0.06,3.02]	0.69
Drink vouchers	97 (89)	100 (83)	96 (92)	99 (94)	0.88 [0.45,∞]	1
Cheap drink promotions (discounted drink promotions, 2 for price 1)	98 (90)	99 (82)	94 (90)	100 (95)	0.91 [0,17.29]	0.95
All you can drink functions	89 (97)	99 (82)	93 (89)	100 (95)	0.91 [0,17.27]	0.95
*Do not conduct any promotions*	76 (70)	92 (76)	68 (65)	92 (87)	0.91 [0.31,-2.66]	0.86

*differing denominator due to missing data

-unable to calculate Odds Ratio (OR) due to both groups achieving 100% at follow-up

### Risky consumption and alcohol related harm

For both complete case and multiple imputation analytical approaches, there were no significant differences between groups on measures of alcohol consumption by members (Table 6). The odds of members from intervention clubs, relative to control clubs, drinking at risky levels at follow-up, ranged from 0.65–0.71, an effect which approached significance. There were no significant differences between members (*N*=34) of intervention clubs that received a full dose of the intervention (*N*=6) compared to those clubs which did not for any of the member outcomes (Graduated Frequency Index *p* = 0.20 and 0.37 respectively and Alcohol AUDIT *p* = 0.88 and 0.86 respectively).

**Table 6:** Risky alcohol consumption and related harm

	Intervention club members		Control club members		Complete Cases [*N* = 1901]		Multiple Imputations [*N* = 2192]	
	*Baseline* (*N* = 515)	*Follow-up* (*N* = 430)	*Baseline* (*N* = 491)	*Follow-up* (*N* = 465)	OR [95% CI]	*p*-value	OR [95% CI]	*p*-value
*Graduated frequency Index*								
Risky drinking at the club at least once a month within the last 3 months (5 or more drinks) % (n)	29 (147)	20 (87)	22 (107)	21 (98)	0.65 [0.41,1.02]	0.06	0.71 [0.45,1.10]	0.13
Risky drinking at the club on at least one occasion in the last 3 months (5 or more drinks) % (n)	47 (243)	38 (164)	43 (212)	36 (166)	0.93 [0.63,1.37]	0.72	0.98 [0.67,1.44]	0.92
*Alcohol AUDIT*								
Risk of alcohol related harm (score > = 8) % (n)	31 (160)	32 (138)	28 (139)	28 (128)	1.07 [0.71,1.61]	0.73	1.12 [0.75,1.67]	0.57
Mean AUDIT score Mean (SD)	6.22 (4.38)	6.18 (4.20)	5.69 (3.78)	5.73 (4.06)	−0.09 [−0.84,0.65]	0.81	−0.04 [−0.75,0.67]	0.91

### Intervention engagement

During the first year of the intervention (2015), 91 (99%) intervention clubs had logged into the web-based program (Table 7). Of those, 78 (85%) completed the annual online assessment. Eight (10%) clubs completed their action plan which was generated from the completed online assessment, and 21 clubs (27%) partially completed their action plan.

**Table 7:** Intervention club (*N* = 92) engagement with web-based program over the 3 year intervention period

Intervention year	Logged into web program % (n)	Completed annual assessment % (n)	Completed action plan % (n)	Partially completed action plan % (n)
2015 (*N* = 92)	99 (91)	85 (78)	10 (8)	27 (21)
2016 (*N* = 92)	88 (81)	88 (72)	75 (54)	15 (11)
2017 (*N* = 87)	80 (74)	81 (60)	73 (44)	1 (6)

In 2016, 81 (88%) intervention clubs logged into the web-based program. Of those, 72 (88%) completed the annual online assessment. Fifty-four (75%) completed their action plan and 11 clubs (15%) partially completed their action plan.

In the final year of intervention (2017) 74 (80%) intervention clubs logged into the web-based program. Of those, 60 (81%) completed the annual online assessment. One club complied with 100% of practices and therefore did not generate an action plan. Of the remaining clubs, 44 (73%) completed their action plan, with 6 (1%) partially completing their action plan.

## Discussion

This large randomised controlled trial is the first to test the impact of a web-based intervention to support the sustained implementation of best-practice alcohol management practices by community sporting clubs. The trial found that practice implementation was sustained over time in both intervention and comparison groups. While there was the suggestion (*p* = 0.06) that risky consumption of alcohol use may have been reduced among members of the intervention, relative to control clubs, there was no significant effect on measures of alcohol intake. The findings provide important information for policy makers and practitioners interested in supporting sustained improvement in alcohol managements in community sporting clubs.

Within both the intervention and control groups there was no attenuation in the implementation of the individual alcohol practices targeted by the intervention. More than 90% of clubs in both groups were meeting more than 10 alcohol practices at follow up. The findings suggest that the implementation support received by intervention or control clubs is sufficient to sustain practice implementation. These conclusions are surprising. as evidence from systematic reviews indicate that sustained implementation is uncommon across a variety of health and community settings [18, 19]. Further, in sporting clubs in particular, significant decay in implementation was expected given the volunteer and transient nature of sporting club officials, the lack of clear governance structures within these organisation and their considerable resource constraints [51–53]. While it is unclear whether and by how much implementation would have eroded had clubs in the control group received no implementation support (rather than usual support provide by the Good Sports program), the findings suggest that once clubs attain high levels of practice implementation, the web-based or usual model of implementation support to maintain such practices is sufficient. The requirement of clubs to progress slowly through each level of the program, taking up to 5 years to reach level three, and the requirement for ongoing accreditation may have been important determinants in the sustained implementation in both groups.

There was a non-significant difference in risky drinking between members of intervention, relative to control clubs at follow-up. The effect size reported on the outcome, however, is similar to that reported in an earlier efficacy trial of the Good Sport program [14] where clubs that had no previous exposure to the program were recruited. There was no difference between groups for other alcohol related harm outcomes where overall mean AUDIT score and risk of alcohol related harm was low at baseline and found to be sustained at follow-up for both groups.

The current study should be considered within the context of its strengths and limitations. Strengths of the study include the randomised controlled design, blinding of outcome assessors, objective collection methods of outcome measures and large sample size. While study attrition was low overall, more intervention than control clubs were lost to follow-up or were ineligible (Intervention clubs *n* = 9; control clubs *n* = 1). One limitation of the study includes the decline in intervention engagement over the 3-year period. There was a 19% drop in clubs logging into the web portal across the 3 years, and a 4% drop in completed annual assessments. This may have reduced the opportunity for an intervention effect. Strategies to increase sustained engagement of clubs with web-based interventions may improve the effects of future trials.

## Conclusions

The study found high rates of sustained program implementation in both the web-based intervention and usual care comparison group. Such findings suggest that once implementation is achieved, high levels of implementation are sustained following either usual or web-based support. The findings provide useful information for policy makers and practitioners for the sustained implementation of programs aimed at reducing alcohol harm in this setting.

## Data Availability

The datasets used and/or analysed during the current study are available from the corresponding author on reasonable request.

## Abbreviations

AUDIT: Alcohol Use Disorder Identification Test
CATI: Computer Assisted Telephone Interview
MI: Multiple Imputation
NSW: New South Wales
OR: Odds Ratio
